# Low Fees and Cheap Stock

**Published:** 1844-12

**Authors:** G. G. Brewster


### ARTICLE VII.

### *Low Fees and Cheap Stock.* By G. G. Brewster, M. D.

There are some new species of fraud developing themselves, of a two flagrant character to pass without exposure to the dental profession, which, I presume you will conceive, deserve some notice in the Journal.

One case came to my observation about two months since. A southern gentleman, who had been travelling north with his lady, had taken occasion to stop at the Tremont House in Boston. Taking up a newspaper, he saw several advertisements of dentists, whose sleight-of-hand operations and liberality in fees induced him to suggest to his lady the economy of turning in the gold plate of teeth she had been wearing during several years, and employ the skill of a low-priced Boston dentist. The lady readily acceded, called on a man of *liberal promise* in the profession, and bargained for a gold plate with six upper mineral teeth, for which she was to give him twelve shillings for each tooth, and plate included, and her own old gold plate to be given to the dentist as a part of the agreement of parties.

The dentist proceeded through the preparatory process. After a convenient time he inserted the plate and teeth, telling the lady of the durability of his work and the unequalled overflow of patronage afforded by the publicity given to his new and original operations, known in the process to himself only. The superiority had already induced a neighboring established dentist to make an offer of a thousand dollars for a disclosure of a discovery and professional invention to facilitate dental operations.

A few days passed away, and the travellers arrived at Portsmouth on their way to Maine. One of the teeth soldered to the lady's plate gave out; the gentleman and lady called at my rooms for the purpose of having the tooth restored. On examing the plate I congratulated the lady for having the finest of gold to compose her plate, and assured her that such pure metal could prove no more injurious to health than it could be unpleasant to her taste. The entire surface presented an uncommonly beautiful gold color.

After matching and fitting a plate tooth, and conducting it through the soldering and cleansing process, I discovered the metal had assumed a white complexion, and ascertained that the entire metallic parts were constituted of SILVER, (not *platina*) excepting the small gold plate and solder which I added to the inside of the new tooth. When the plate came into my room it was *gilded*. The application of heat removed the gold surface and divulged the miserable secret. The nefarious man who was guilty of this trick is one of the *eighteen-pence tooth-pulling class of operatives*, whose names are haloed by the bombulations of newspaper-puffs, and acknowledged by dental surgeons as only catch-penny mountebanks, without a claim on the respect of honorable practitioners.

